# The placenta findings from an XYY abortus: a case report

**DOI:** 10.1186/1752-1947-7-228

**Published:** 2013-10-01

**Authors:** Rong Fan

**Affiliations:** 1 Department of Pathology and Laboratory Medicine, Indiana University School of Medicine, Riley Hospital for Children at IU Health, 705 Riley Hospital Dr, Suite 2536, Indianapolis, IN 46202-5200, USA

## Abstract

**Introduction:**

The placenta morphology from an XYY pregnancy abortus has not been reported in the medical literature. This case report consists of the first detailed documentation. The reported case is also highly unusual because the mother had two prior pregnancies with fetuses being confirmed to have Zellweger syndrome and one prior molar pregnancy.

**Case presentation:**

A 43-year-old Caucasian woman presented for induction of labor secondary to diagnosis of XYY chromosomes by chorionic villus sample.

**Conclusions:**

This is the first detailed observation of placenta morphology in an XYY abortus. Although not highly specific, the observation is very unique and should prompt further investigation of karyotyping of the fetus or infant because an XYY individual may be viable and grow to adulthood. The association of an XYY abortus and prior pregnancies with Zellweger syndrome and one prior molar pregnancy is also highly notable.

## Introduction

XYY syndrome is well known and well reported in the medical literature, but its placenta morphology has not been specifically described in the past. We report a case of two Zellweger syndrome fetuses, one molar and one XYY, from a single mother. This is highly unusual and, to the best of our knowledge, has not been previously reported.

## Case presentation

A 43-year-old Caucasian woman, gravida 4, para 0-2-1-0, presented for induction of labor secondary to diagnosis of XYY chromosomes found on a chorionic villus sample at 16+2 weeks by a first-trimester ultrasound. She denied contractions, leakage of fluid, or vaginal bleeding. Her past medical history was complicated by two previous abnormal pregnancies. Her first child had Zellweger syndrome, was delivered preterm at 36 weeks, and died at 4 years. The diagnosis of Zellweger was made first through biochemical testing of the child’s blood and then confirmed by molecular studies through a research protocol, which revealed two mutations.

Her second pregnancy ended with an induction of labor at 27 weeks due to Zellweger syndrome. She also has had a molar pregnancy. Her husband’s family history is only remarkable for his brother having an apparently isolated congenital heart defect.

Gross examination of her placenta revealed a small placenta of 60g, 9.0 × 8.4 × 2.2cm placental disc with attached membranes and umbilical cord. The membranes were tan, semitranslucent with a marginal (50%) to circummarginate (50%) insertion. The point of rupture was indeterminate. The 13.5 × 0.5cm, two-vessel umbilical cord inserted eccentrically, 0.8cm from the disc margin. The fetal surface was purple–pink with small caliber vessels and minimal subchorionic fibrin deposition. The maternal surface was red–pink, and spongy with poorly formed, markedly disrupted lobules. Sectioning revealed a tan–pink, soft, homogeneous parenchyma with no discrete lesions.

**Figure 1:**
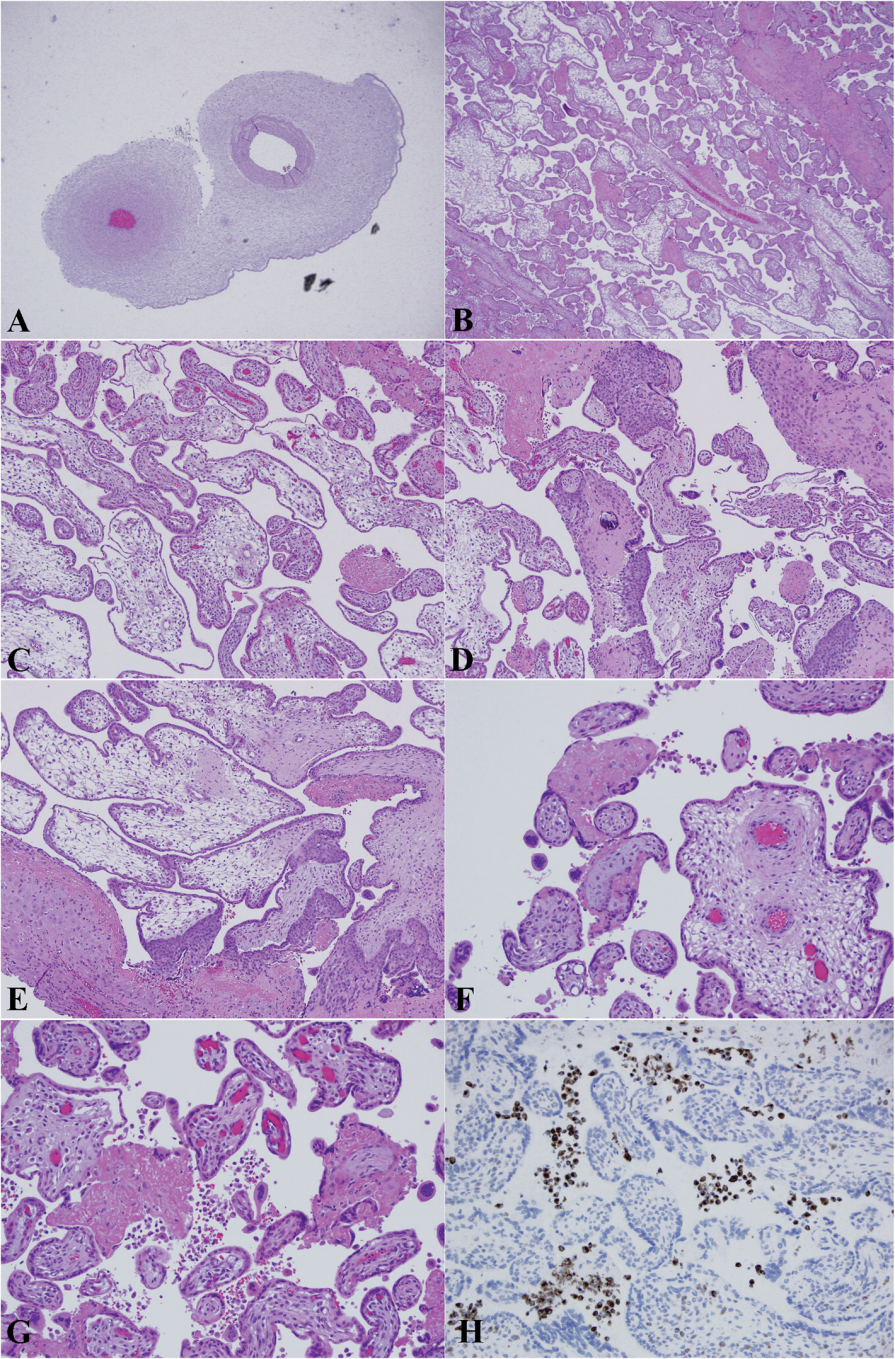
**The spectrum of placenta morphology in XYY abortus. (A)** Umbilical cord showed two vessels on cross-section. **(B)** Histological section of placenta parenchyma at lower magnification. **(C** and **D)** Medium power microscopic examination revealed frequent syncytial budding, villous contours with excessive scalloping. **(E** and **F)** High magnification showing similar features of different fields; note the villi were immature and sparsely vascularized. **(G)** Patchy increased intervillous inflammatory infiltrates. **(H)** These infiltrates were CD68 positive, confirming the diagnosis of chronic intervillositis. The association of patchy chronic intervillositis is thought to be incidental.

Microscopically, a monoarterial umbilical cord was confirmed. The chorionic villi morphology was remarkable for edema and/or hydropic, marked size variation, frequent syncytial budding and, most strikingly, many featuring very irregular villi contours. The extensive villi contour variation, some of which could be described as scalloping, was accompanied by minimal elevation of perivillous fibrin deposition. The circumferential trophoblastic hyperplasia was also minimal. Minor foci of microcalcification were noted. Also of note, was a patchy increase of prominent mononuclear inflammatory cell infiltrate in the intervillous space consistent with chronic intervillositis, confirmed by the immunohistochemistry staining of CD68. The spectrum of microscopic appearance is shown in Figure 1.

A cytogenetic study for the current pregnancy from the chorionic villus specimen was performed. Of the 21 counted cells, eight cells were analyzed, and four karyotypes were prepared from G-banded slides at an average band level of 463 bands. Cytogenetic studies showed that this fetus had evidence of two cell lines. The first line demonstrated a karyotype of 47,XYY. The second line demonstrated a karyotype of 47,XYY,del (10)(q10). In the nonmosaic form, deletion of the entire long arm of chromosome 10 is incompatible with survival to term. However, in this case, del(10q) was dismissed as a culture artifact. Hence, her fourth and current pregnancy was complicated by XYY syndrome. The fetus was predicted not to be affected by a Zellweger spectrum disorder. This conclusion was based on the presence of the maternal mutation (c.764dupA) and the absence of the paternal mutation (c.928C>G;p.His310Asp). An induction of labor and termination of pregnancy was performed as per the patient’s request.

## Discussion

The XYY syndrome is observed in about 1 in 1000 male births. These individuals typically have tall stature and average intelligence. Cognitive or behavioral phenotypes, including developmental delay, learning disabilities, hyperactivity, and attention problems, may be present [1]. Reportedly correctional institutions tend to have more of this chromosome abnormality in their populations [2]. Although testicular function, testosterone levels, and fertility are usually normal, patients may have hypogonadism and sterility.

The Zellweger syndrome spectrum is a continuum of three phenotypes (Zellweger syndrome, neonatal adrenoleukodystrophy, and infantile Refsum disease) due to abnormalities of peroxisome biogenesis [3,4]. These conditions are inherited in an autosomal recessive pattern with a 25% risk for recurrence in subsequent pregnancies. Because this pregnancy was not complicated by Zellweger syndrome, the placenta morphology is considered to be purely attributed to XYY karyotype. The role of chronic intervillositis in XYY syndrome is not entirely clear. This association is considered incidental at this point, although it is known to be associated with adverse pregnancy outcomes and the risk of recurrence in subsequent pregnancies [5].

The placenta morphology in XYY syndrome has not yet been reported in the medical literature. However, studies from some general placenta aneuploidy studies revealed similar observations such as extensive scalloping, increased ramification of the main villous trunks, and increased syncytial budding [6,7], but some placenta morphology in these aneuploidy cases may be secondary to the gestation age or reduction in the villous circulation due to a cardiovascular defect. The secondary changes may include trophoblastic hypoplasia, stromal edema, cavitation, or reduced vascularization. In addition, most study cases were trisomy 21, trisomy 18 [8] and no XYY karyotype cases were included. Furthermore, the consistency of the observation was not good. Trisomy 18 has smaller villus diameter and reduced number of capillaries per villus cross-section. The trisomy 21 group is said to have villi with increased percentage of two-layered trophoblasts present and an increased proportion of villus capillaries [8]. These observations and some other observations such as basophilic stippling of the basement membrane and increased intervillous fibrin deposition are prone to observer bias and gestational age of the sample placentas.

## Conclusions

In summary, this is the first detailed observation of placenta morphology of an XYY abortus. The major observations include small placenta, two-vessel umbilical cord, frequent syncytial budding, striking villous contours, and variation in many areas. This case is also highly unusual because the mother had two prior pregnancies with the fetuses being confirmed to have Zellweger syndrome and one prior molar pregnancy.

## Consent

Written informed consent was obtained from the patient for publication of this case report and accompanying images. A copy of the written consent is available for review by the Editor-in-Chief of this journal.

## Competing interests

The author declares that he has no competing interests.
